# Resveratrol: A miraculous natural compound for diseases treatment

**DOI:** 10.1002/fsn3.855

**Published:** 2018-10-26

**Authors:** Mehdi Koushki, Nasrin Amiri‐Dashatan, Nayebali Ahmadi, Hojjat‐Allah Abbaszadeh, Mostafa Rezaei‐Tavirani

**Affiliations:** ^1^ Department of Biochemistry Faculty of Medicine Tehran University of Medical Sciences Tehran Iran; ^2^ Student Research Committee Proteomics Research Center Shahid Beheshti University of Medical Sciences Tehran Iran; ^3^ Proteomics Research Center Faculty of Paramedical Sciences Shahid Beheshti University of Medical Sciences Tehran Iran; ^4^ Hearing Disorders Research Center Shahid Beheshti University of Medical Sciences Tehran Iran

**Keywords:** antioxidant, dietary, natural compound, polyphenol, resveratrol

## Abstract

Resveratrol (3, 5, 4′‐trihydroxystilbene) is a nonflavonoid polyphenol that naturally occurs as phytoalexin. It is produced by plant sources such as grapes, apples, blueberries, plums, and peanut. This compound has critical roles in human health and is well known for its diverse biological activities such as antioxidant and anti‐inflammatory properties. Nowadays, due to rising incidence of different diseases such as cancer and diabetes, efforts to find novel and effective disease‐protective agents have led to the identification of plant‐derived compounds such as resveratrol. Furthermore, several in vitro and in vivo studies have revealed the effectiveness of resveratrol in various diseases such as diabetes mellitus, cardiovascular disease, metabolic syndrome, obesity, inflammatory, neurodegenerative, and age‐related diseases. This review presents an overview of currently available studies on preventive properties and essential molecular mechanisms involved in various diseases.

## 1 INTRODUCTION

**Figure 1:**
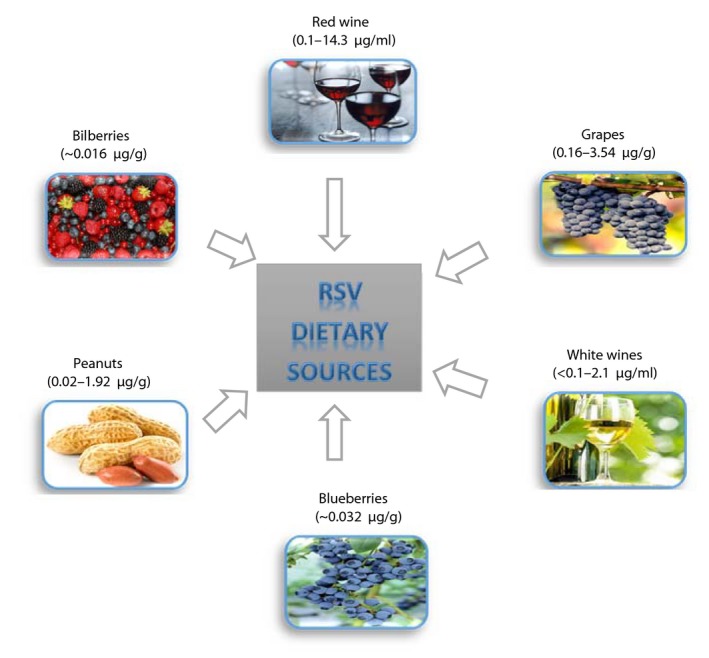
Dietary sources of resveratrol

**Figure 2:**
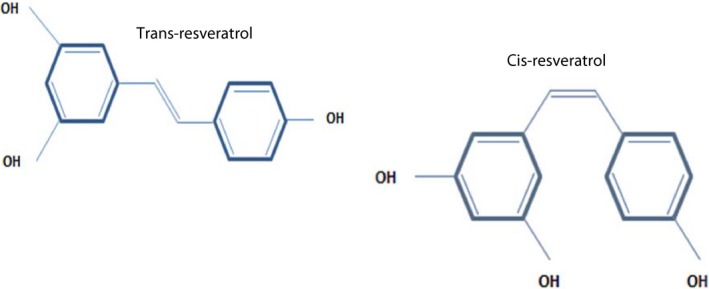
Chemical structure of resveratrol. (a) Trans‐resveratrol, (b) Cis‐resveratrol

Resveratrol (RSV) (3, 5, 4′‐trihydroxystilbene) is a phenolic micronutrient compound that is formed naturally by 70 different plant species such as grapes, berries, peanuts, and pines (Harikumar & Aggarwal, 2008; Shishodia & Aggarwal, 2006). The major RSV dietary sources are shown in Figure 1. This metabolite was initially recognized as an antibiotic produced in response to various environmental stresses, such as mechanical damage, microbial infection, UV radiation, heat, and pathogenic conditions (Harikumar & Aggarwal, 2008; Pervaiz, 2003). It was initially extracted from the roots of white hellebore (*veratrum grandiflorum O. Loes*.) by Michio Takaoka in 1940 (Saiko, Szakmary, Jaeger, & Szekeres, 2008). The plants have varying potential for RSV production. For example, RSV concentration in grapes and peanuts is estimated to be in the range of 0.16–3.54 and 0.02–1.92 μg/g, respectively. The highest levels of RSV are found in *Polygonum cuspidatum* (524 μg/g; also known as *Fallopia japonica*, Japanese knotweed), which has been used in traditional Asian medicine for inflammatory treatments (Catalgol, Batirel, Taga, & Ozer, 2012; Kukreja, Wadhwa, & Tiwari, 2013). RSV has a stilbene structure, consisting of two aromatic rings connected by a methylene bridge. Two isomeric forms (*cis* and *Trans*) of RSV exist in nature; however, only the trans form of RSV participates in the majority of biological activities because of its stability (Wu & Liu, 2013) (Figure 2). Despite 70% absorption of resveratrol in the body (Walle, 2011), studies performed on rats showed that its bioavailability is relatively low due to its rapid metabolism in the intestine and liver (Goldberg, Yan, & Soleas, 2003). RSV bioavailability exhibits a variability from one person to another that depends on age and gender (Nunes et al., 2009). Bode et al. (2013) reported that RSV metabolism by human gut microbiota shows pronounced interindividual diversity based on the study of health‐related effects of this compound. Several RSV‐derived metabolites also have been reported in the plasma and urine of animals and humans (Azorín‐Ortuño et al., 2011). However, the activity of all these metabolites is not clear. Studies performed on animal models to date have shown no apparent adverse effects of RSV (Johnson et al., 2011; Patel et al., 2010; Rotches‐Ribalta, Andres‐Lacueva, Estruch, Escribano, & Urpi‐Sarda, 2012). The only reported side effect in the studies was the onset of diarrhea after receiving 200 mg of resveratrol daily, which was not associated with clinical complications (la Porte et al., 2010). Overall, adverse effects of RSV have been minor and it appears to have a good safety. RSV causes human platelets aggregation in vitro (Bertelli et al., 1996), therefore recommended not to intake with anticoagulants and antiplatelet drugs (Abu‐Amero, Kondkar, & Chalam, 2016). Nowadays, RSV attracts increasing attention due to naturally occurring compound and the wide range of biological activity and preventive effects on different diseases such as cancers (Gusman, Malonne, & Atassi, 2001; Savouret & Quesne, 2002), neurodegenerative diseases (Chang et al., 2012), cardiovascular disease (Cao & Li, 2004), anti‐inflammatory (Donnelly et al., 2004; Koushki, Dashatan, & Meshkani, 2018), and antioxidant activities (Kawada, Seki, Inoue, & Kuroki, 1998). In addition to RSV, different oligomers (Lins, Ribeiro, Gottlieb, & Gottlieb, 1982) of this compound were found to exhibit broadly biological activities, such as antiviral, anti‐fungal, antibacterial, and anticancer activities (Li, Henry, & Seeram, 2009; Zain, Ahmat, Norizan, & Nazri, 2011). The overall aim of this study was to reveal the potential beneficial effects of resveratrol and its therapeutic mechanisms in various diseases.

## 2 RESVERATROL AND DISEASES

### 2.1 Resveratrol as antidiabetic compound

Diabetes mellitus is a metabolic disease, which affects approximately 5% of people worldwide. Type 1 and type 2 are the most common types of diabetes. Diabetes is associated with metabolic abnormalities and long‐term complications such as antipathies, cardiovascular disorders, retinopathy, renal disorder, and neuropathies. On the other hand, despite many attempts in the management of diabetes, current therapeutic methods are accompanied by side effects. Recent investigation indicates that resveratrol is a natural compound exerting numerous beneficial effects on diabetes, which are extensively studied in animal models and in diabetic humans.

#### 2.1.1 Resveratrol and type 1 diabetes

**Figure 3:**
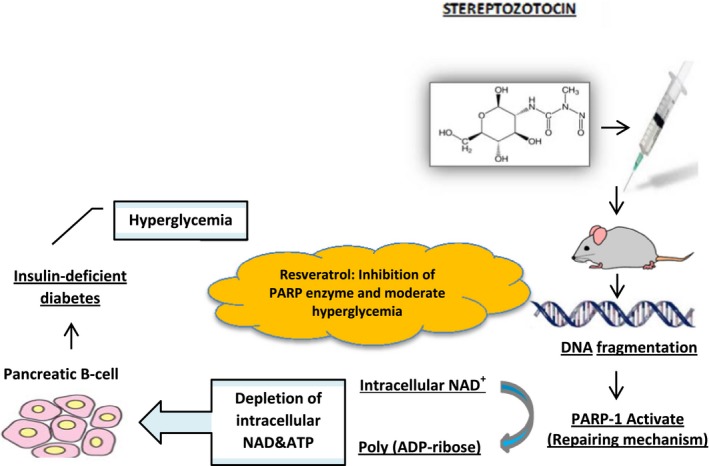
Resveratrol effect in streptozotocin‐induced diabetic rat models

**Figure 4:**
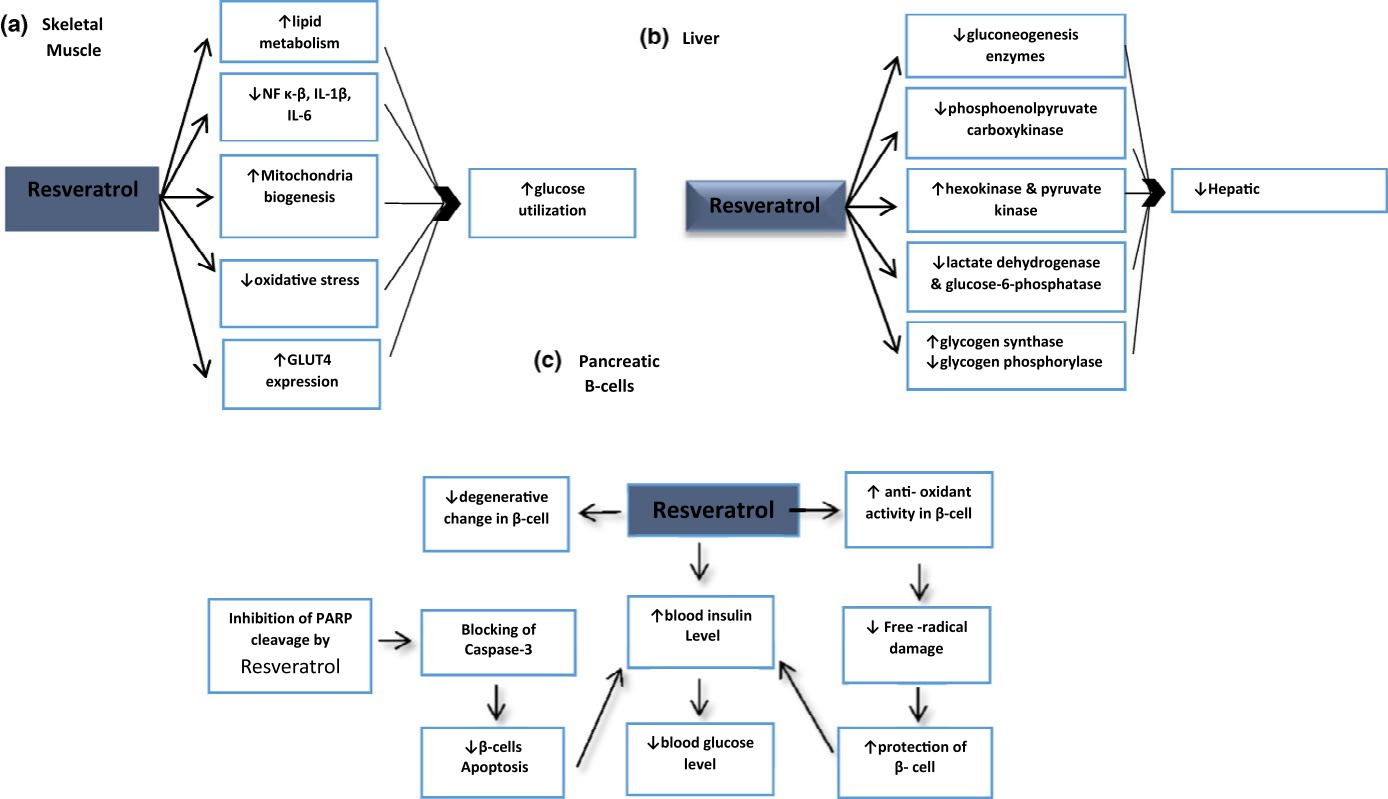
Effective mechanisms of resveratrol in experimental type 1 diabetic models in (a) skeletal muscle, (b) liver, and (c) pancreatic B cells. PARP: poly (ADP‐ribose) polymerase‐1

Type 1 diabetes, known as insulin‐dependent diabetes, is a chronic condition that results from the autoimmune destruction of beta cells of the pancreas that produce insulin, a hormone needed to allow sugar (glucose) to enter the cells to produce energy. Only 5%–10% of people with diabetes suffer from this type of disease. Maintaining normoglycemia and preservation of pancreatic β‐cells are the main management aspects in type 1 diabetics (Szkudelski & Szkudelska, 2015). Animal studies clearly confirmed anti‐hyperglycemia effects of RSV and its pancreatic β‐cell protection. The effects of resveratrol in decreasing blood glucose were found in rats with diabetes induced by streptozotocin (STZ) alone and STZ with nicotinamide (NA) (Palsamy & Subramanian, 2010; Silan, 2008). The results of the previous studies have shown that RSV could maintain normoglycemia and protect of pancreatic β‐cells in type 1 diabetics. The normal glucose level in blood is preserved by insulin. Since pancreatic beta cells are the only source of this hormone, it can be concluded that the RSV pancreatic β‐cell protection property is promising. Actually, RSV improves antioxidant capacity in pancreatic beta cells by increasing the antioxidant enzyme content of the cells including superoxide dismutase, catalase, and glutathione S‐transferase. Hereby, pancreatic tissue is protected from the free radical compounds (Palsamy & Subramanian, 2010). In addition, RSV inhibits PARP enzyme cleavage, because of its caspase‐3 enzyme activity blockage and hence decreasing apoptosis of beta cells in STZ‐induced and STAZ‐NA‐induced degeneration of the cells (Figure 3). Protection of beta cells from apoptosis led to blood insulin‐level promotion and decreasing of blood glucose level in type 1 diabetes (Ku et al., 2012). Another main action of RSV compound can be explored in type 1 diabetic skeletal muscle. It was demonstrated that RSV alleviates skeletal muscle dysfunction in type 1 diabetic animal models (Chang et al., 2014; Chen et al., 2011). One of the RSV recovering mechanisms in skeletal muscle pathological conditions is mitochondrial biogenesis stimulation and increasing of lipid metabolism in diabetic models. According to Chen et al. (2011) study, RSV also exerts anti‐inflammatory effects and decrease NF‐κB, IL‐1β, and IL‐6 cytokines. Oxidative stress is another contributing factor to myopathy co‐occurring with inflammation in type 1 diabetes (D'Souza, Al‐Sajee, & Hawke, 2013). Data confirmed the effect of RSV on oxidative stress reduction in skeletal muscle (Chang et al., 2014). It is noteworthy that RSV affects skeletal muscles by increasing GLUT4 expression and augmenting intracellular glucose transport (Deng, Hsieh, Huang, Lu, & Hung, 2008). RSV was also found to reduce gluconeogenesis activity and increase liver glycogenesis, which leads to hepatic glucose output reduction. It should be noted that effects of RSV in the liver are originated from increasing blood insulin level. In addition, RSV alleviated liver tissue abnormalities by hepatoprotective effects (Hamadi, Mansour, Hassan, Khalifi‐Touhami, & Badary, 2012). Overall, the anti‐diabetic effects of resveratrol well established in diabetic animals (Chang et al., 2011, 2014; Hamadi et al., 2012; Ku et al., 2012). Some of the possible mechanisms of resveratrol on type 1 diabetic animal models are shown in Figure 4.

#### 2.1.2 Resveratrol and type 2 diabetes

**Figure 5:**
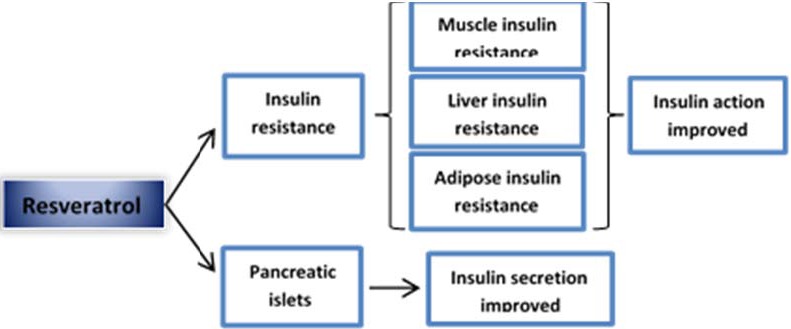
Effects of resveratrol on insulin resistance. Animal studies indicate that resveratrol improves insulin action in various models of insulin resistance. Resveratrol‐induced decrease in insulin resistance is known to result from changes in skeletal muscle, the liver, and adipose

Type 2 diabetes is the most common form of diabetes. About 90% of all diabetic patients suffer from type 2 diabetes. In these patients, body resists the effects of insulin hormone or shows an insulin secretion deficiency. It is recently established that factors such as inflammation and oxidative stress contribute to worsening of insulin resistance and to β‐cell failure in type 2 diabetics (Hamadi et al., 2012). Besides, insulin resistance in adipocytes, hepatocytes, and muscle cells is still complicating issue. In this context, animal studies provided evidence that RSV as a natural compound has beneficial effects on both mechanisms in these tissues and pancreatic β‐cells (Jiang et al., 2013; Palermo, Maggi, Maurizi, Pozzilli, & Buzzetti, 2014). Although the effects of resveratrol on the inflammatory responses have been investigated in different models, this function of resveratrol in skeletal muscle cells remains unknown. In Sadeghi et al. study, the plausible role of resveratrol in ameliorating palmitate‐induced inflammation in C2C12 skeletal muscle cells investigated. The results of this study indicated that anti‐inflammatory effects of resveratrol are more likely to be a Sirtuin‐1 (SIRT‐1)‐independent mechanism in skeletal muscle cells (Sadeghi, Seyyed Ebrahimi, Golestani, & Meshkani, 2017). The results of several studies confirmed the SIRT‐1 and AMP‐activated protein kinase (AMPK) as the main molecular targets for RSV to protect against pancreatic β‐cell dysfunction and improving insulin sensitivity (Do et al., 2012; Guo et al., 2014). Actually, activation of these targets by RSV as a drug led to cellular and enzymatic phenomena including decreased lipid accumulation, inflammation, and oxidative damage in liver, muscle, and adipocyte tissues and finally led to insulin action improvement in type 2 diabetes (Figure 5). However, another possible mechanism suggested in different studies including increased phosphorylation of insulin receptor, IRS‐1 expression, and phosphorylation of Akt (Chen, Li et al., 2012; Deng et al., 2008). In another in vivo study conducted by Cheng et al., it has been shown that resveratrol promoted nuclear factor erythroid 2‐related factor‐2 (Nrf2) phosphorylation, in the pancreas of methylglyoxal‐treated mice. This finding supports that resveratrol may be useful in the treatment of type 2 diabetes by protecting against pancreatic cell dysfunction (Cheng, Cheng, Lee, Chung, & Chang, 2015). Oxidative stress is also another factor leading to β‐cell failure in type 2 diabetes, and according to animal investigations, RSV decreases the antioxidant damage of β‐cells. Although the exact mechanism of action is not clearly identified, evidences show that this compound is able to improve insulin sensitivity in insulin resistance condition in different animal models. In animal model of type 2 diabetes (db/db mice) with decreased pancreatic β‐cell mass, RSV was found to improve islet function, reduce oxidative stress, and decrease islet destruction and degenerative changes (Do et al., 2012; Lee et al., 2012). Moreover, RSV partially prevents β‐cell failure and increases β‐cell mass. In general, the management of diabetes involves three main aspects: reduction in blood glucose, preservation of beta cells, and, in the case of type 2 diabetes, improvement of insulin action. Data from the literature indicate that the beneficial effects of RSV in relation to diabetes comprise all these aspects.

### 2.2 Resveratrol and cardiovascular diseases

**Figure 6:**
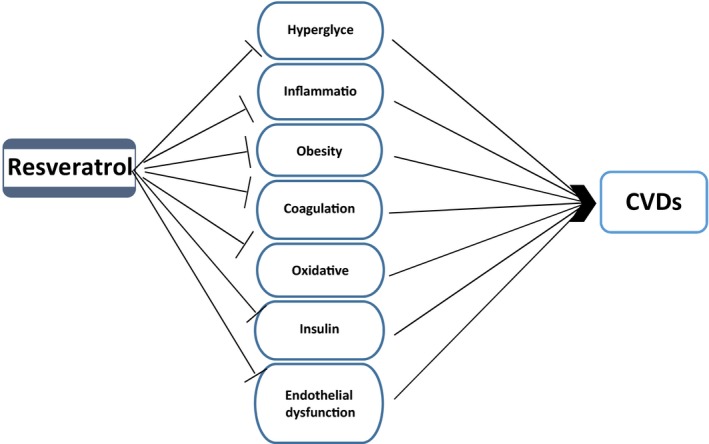
Targets of resveratrol in cardiovascular disease. Resveratrol, by interrupting these factors and events, may be possible to prevent or slow the development of cardiovascular disease

**Figure 7:**
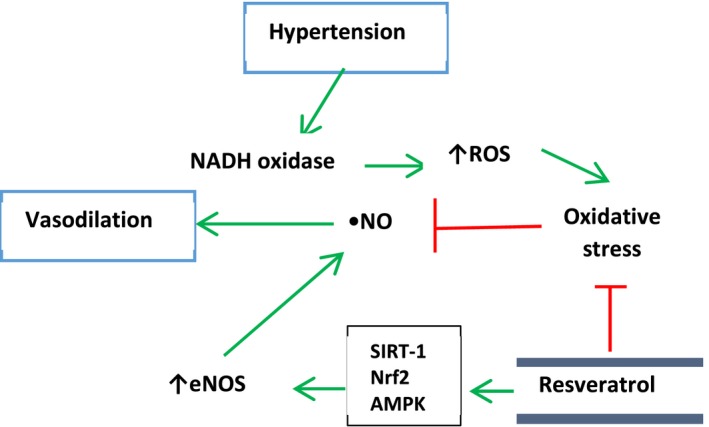
Mechanisms involve in hypertensive properties of resveratrol. Also, endothelium‐independent mechanisms have been reported such as AMP‐activated protein kinase activation which leading to inhibition of angiotensin II‐induced phosphorylation of Myosin light chain (Cao, Fang, Xia, Shi, & Jiang, 2004)

Because of increased incidence of cardiovascular diseases (CVDs) including atherosclerosis, hypertension, stroke, ischemic heart disease, and heart failure, which are the major cause of mortality and morbidity in the world, more investigations are underway in this field (Laslett et al., 2012). Potential cardio‐protective role of RSV against CVDs has also been suggested by studies (Figure 6). Several studies have been shown anti‐atherosclerotic, anti‐hypertensive, anti‐myocardial ischemia, anti‐stroke, and heart failure effects of it. Improvement of bioavailability of nitric oxide (NO) by RSV is one of the cardio‐protective mechanisms of this compound (Leikert et al., 2002). NO contributes to improved vasodilation and decreased platelet aggregation, leukocyte recruitment, and proliferation of smooth muscle cells, which are inhibitors of atherosclerosis formation and progression (Li & Förstermann, 2000). In general, the beneficial effects of RSV via oxygen‐derived radical scavenging or by increasing NO bioavailability in vitro could be promising against CVDs (Frombaum, Le Clanche, Bonnefont‐Rousselot, & Borderie, 2012). In the case of anti‐atherosclerotic effects of RSV, atherosclerosis is now may be considered as an inflammatory disease. Since low‐density lipoproteins (LDLs) involved in atherosclerosis, improvement of lipid profile by RSV, could be an anti‐atherosclerotic potential therapeutic agent. Several preclinical studies have shown that RSV could affect lipid profile significantly via decreasing plasma triglyceride content, LDL cholesterol, and also increasing HDL cholesterol levels (Göçmen, Burgucu, & Gümüşlü, 2011). Cho, Ahn, Kim, Choi, and Ha (2008) reported RSV effects on hypocholesterolemia by down‐regulation of 3‐hydroxyl‐3‐methylglutaryl‐COA reductase enzyme (HMG‐CoA) reductase enzyme in cholesterol biosynthesis processes. On the other hand, also increasing of LDL receptors expression by RSV in hepatocytes under in vitro conditions could led to decreasing of blood LDL cholesterol levels (Yashiro, Nanmoku, Shimizu, Inoue, & Sato, 2012). In addition, RSV decreases LDL oxidation as one of the key events involving in the atherogenesis. Decreasing of LDL oxidation is the result of antioxidant property of RSV, which leads to improvement of lipid metabolism (Witztum & Steinberg, 1991). The cross‐talk between β‐catenin/Wnt and forkhead box O (FOXO) pathways was impaired in peripheral blood mononuclear cells (PBMCs) of coronary artery disease (CAD) patients. In a recent study, Shanaki et al. performed a case–control study in coronary artery disease and demonstrated that RSV treatment could lead to a modest increase in the manganese superoxide dismutase (MnSOD) activity independent of β‐catenin/FOXO pathway. Despite a modest improvement in the β‐catenin/FOXO pathway after RSV treatment, this pathway was not completely repaired in CAD patients (Shanaki et al., 2016). All of these properties suggest that RSV could exert its effects on important factors involved in the atherosclerotic process. It exhibits its beneficial effects on CVDs via multiple functions including activation SIRT‐1, nitric oxide synthase (eNOS), and Nrf2 and decreases tumor necrosis factor‐α (TNF‐α) production (Li, Xia, & Förstermann, 2012). Hypertension is one of main factors for CVDs (Rivera, Morón, Zarzuelo, & Galisteo, 2009), and anti‐hypertensive effects of RSV have been shown in some animal studies (Dolinsky et al., 2013). The mechanism of RSV against hypertension includes endothelium‐dependent pathways (Zordoky, Robertson, & Dyck, 2015). In this mechanism, it increases AMPK, Nrf2, and SIRT‐1 factors, which finally lead to improvement of flow‐mediated vasodilation in animal models (Figure 7). In addition, RSV has effect on myocardial ischemia. Inhibition of platelet aggregation and a potential regeneration of tissue in the infarcted area are some RSV effects on myocardial ischemia (Raj et al., 2014). One of protective mechanisms by RSV in cardiomyocytes is induction of autophagy via SIRT‐1 and AMPK activation and inhibition of fractalkine protein as inhibitor of RSV‐induced autophagy (Xuan et al., 2012). Both antioxidant activity and increasing of nitric oxide bioavailability actions of RSV are required to exert its anti‐ischemic effects (Leikert et al., 2002). Studying of cardiac stem cells demonstrated that RSV could potentiate the regeneration of infarcted myocardium (Gurusamy, Ray, Lekli, & Das, 2010). Recently, several lines of evidences have shown the role of micro‐RNAs (miR‐21, miR‐20b, miR‐27a, miR‐9, miR‐29) in cardio‐protective effects of RSV (Mukhopadhyay, Pacher, & Das, 2011).

### 2.3 Resveratrol and neurodegenerative disease

#### 2.3.1 Resveratrol and Alzheimer's disease

Alzheimer's disease (AD) is a neurodegenerative disorder and by far the most common cause of dementia. AD is defined by characteristic pathological features such as deposition of amyloid beta (Aβ) peptides and aggregation of hyper‐phosphorylation of tau protein in the form of neurofibrillary tangles (NFTs) (Selkoe, 2001). Aβ is a 39–43 amino acid peptide fragment derived from APP (amyloid precursor protein) processing by beta (β) and gamma (γ)‐secretase enzymes (Huang, Lu, Wo, Wu, & Yang, 2011). Aβ accumulation leads to a characteristic of AD, that is, progressive loss of neurons and neurological decline (Roberson & Mucke, 2006). Numerous studies yet have been underway to discover the potential improving effects of plant extracts in AD under in vivo conditions (Kashani, Tavirani, Talaei, & Salami, 2011; ; Soheili, Tavirani, & Salami, 2015). Studies have shown that resveratrol as a natural supplement was involved in various pathophysiologic cycles of AD (Baur & Sinclair, 2006). Antioxidant, anti‐amyloidogenic properties, and beneficial effects of resveratrol against neuro‐inflammation are promising in AD prevention. Oxidative stress by an excessive production of ROS in the brain has been strongly involved in the pathogenesis of AD. Because of low contents of antioxidants, the greater rate of oxygen consumption, and higher content of peroxidation of fatty acids, brain tissue is more susceptible to oxidative stress (Romano, Serviddio, de Matthaeis, Bellanti, & Vendemiale, 2009). Increasing the levels of ROS and reactive nitrogen species can led to protein damage, lipid peroxidation, and finally loss of membrane integrity. All of these events cause injury to neural membranes, cellular damage, and memory impairment (Abolfathi, Mohajeri, Rezaie, & Nazeri, 2012). Numerous studies have indicated that ROS increases Aβ production, which in turn induces oxidative stress, accelerating AD progression (Li, Gong, Dong, & Shi, 2012). Antioxidants have been shown to protect against Aβ‐induced neurotoxicity. RSV as an antioxidant reduces iNOS levels and lipid peroxidation in neuronal cells and increases the production of hem oxygenase‐1 (HO‐1) to weaken oxidative damage to the cells (Ma, Tan, Yu, & Tan, 2014). Also, several in vivo and in vitro studies suggested that activation of astrocytes and microglia affects Aβ accumulation leading to secretion of pro‐inflammatory agents such as cytokines (IL‐6, IL‐1β, TNF‐α) and free radicals. Pro‐inflammatory cytokines in turn induce Aβ aggregation causing ROS and TNF production (Capiralla, Vingtdeux, & Zhao, 2012). RSV decreases pro‐inflammatory mediator's production, clears Aβ aggregation, and relieves oxidative stress and neuron cell death (Saiko et al., 2008). In general, numerous studies show that RSV has intense neuroprotective properties in several models, both in vitro and in vivo (rat models) (Table 1). In addition to its monomers, several oligomers of RSV have been developed and thereby could be used as agents in the treatment of AD (Rivière et al., 2010). In addition, Lu and colleagues designed a novel series of RSV derivatives for AD treatment purposes (Lu et al., 2013). Nevertheless, there is not a complete large‐scale clinical trial until yet.

**Table 1:** Some of resveratrol neuroprotective effect studies result in vivo and in vitro in recent years (after 2010)

Sample	Resveratrol effect	Reference
The transgenic *Caenorhabditis elegans* strain CL2006	Aβ aggregation↓	Regitz, Fitzenberger, Mahn, Dußling, and Wenzel (2016)
	Aβ‐induced toxicity↓	
	Increased flux of proteins through the autophagy pathways and actiated proteasomal degradation↑	
PC12 cells	Aβ‐induced apoptosis 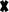	Feng et al. (2013)
	SIRT‐1↑	
	ROCK1↓	
SAMP8 mice	SIRT‐1 and AMPK↑	Porquet et al. (2013)
	Acetylated P53↓	
APP/PS1 mice (diet + resveratrol)	Activated microglia↓	Capiralla, Vingtdeux, Zhao, et al. (2012)
RAW264.7 cells	Phosphorylated IKKα, IκBα, and NF‐κB↓	Capiralla, Vingtdeux, Venkatesh, et al. (2012)
BV‐2 cells	STAT1 and STAT3 activation 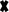	
Ba/F3 cells	Expression of iNO and COX‐2↓	
Neocortical neurons—SAMP8 mice	SIRT‐1 expression↑	Cristòfol et al. (2012)
	Oxidative damage↓	
C67BL/6J mice (diet + resveratrol)	Serum TNF‐α↓	Jeon et al. (2012)
Senescence accelerated mice	Lipid peroxidation↓	Liu, Zhang, Yang, and He (2012)
	Mitochondrial deletion 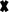	
Sprague‐Dawley rat (oral administrating of resveratrol)	BDNF expression↑	Rahvar et al. (2011)
SH‐SY5Y neuroblastoma cells	Generations of Aβ‐Fe, Aβ‐Cu, and Aβ‐Zn↓	Granzotto and Zatta (2011)
Sprague–Dawley rats	Lipid peroxidation↓	Huang et al. (2011)
	Aβ aggregation in hippocampus↓	
H‐SY5Y human neuroblastoma	Aβ toxicity↓	Granzotto and Zatta (2011)

*Notes*

BDNF: brain‐derived neurotrophic factor; ↓: decrease or down‐regulation; ↑: increase or up‐regulation; inhibition.

#### 2.3.2 Resveratrol and Parkinson disease

Parkinson disease (PD) is a chronic and progressive neurodegenerative disease associated with impairment of motor functions (Sun, Wang, Simonyi, & Sun, 2010). It is the second most common neurodegenerative disease and about 2% of people over the age of 65 suffer from the disease (de Rijk et al., 1997). This disease is an autosomal dominant disorder, and mutations in the alpha‐synuclein gene are the cause of this disease. Although environmental and genetic factors contribute to PD development, the exact etiology remains unclear yet (Gasser, 2005; Sun et al., 2010). Recent studies showed that neurotoxic agents are possible inducers of PD such as manganese (Sun, Yang, & Kim, 1993), dimethoxy phenyl‐ethylamine (Koshimura et al., 1997), paraquat (Miller, Sun, & Sun, 2007), and 1‐methyl‐4‐phenyl‐1, 2, 3,6‐tetrahydropyridine (MPTP) causing oxidative stress, and also mitochondrial dysfunction (Alvira et al., 2007) and inflammation are important factors in dopaminergic neurons degeneration in PD (Giasson, Ischiropoulos, Lee, & Trojanowski, 2002; Sun et al., 2010). Mitochondrial dysfunction plays an important role in the pathogenesis of PD. Production of free radicals occurs in complex I inhibition, and then, oxidative stress could lead to PD (Alvira et al., 2007). Several lines of evidence showed the improvement of mitochondrial respiratory capacities by RSV treatment. In fact, RSV activates SIRT1‐AMPK pathway and then induces PGC‐1alpha activity (Ferretta et al., 2014). PGC‐1alpha activation leads to mitochondrial biogenesis and amelioration of its function. Studies in PD experimental models have shown that antioxidant property of RSV protects dopaminergic neurons (Blanchet et al., 2008). Moreover, RSV‐mediated activation and expression of SIRT‐1 reduce alpha‐synuclein aggregation in PD (Donmez et al., 2012). SIRT‐1 deacetylates and activates heat shock factor 1 (HSF1) affecting heat shock protein 70 (HSP70) transcription and the latter can decrease abnormal protein aggregation (Westerheide, 2013). In summary, many environmental factors can activate oxidative pathway, which causes neurodegeneration. On the other hand, RSV has been shown to suppress oxidative pathway (Saiko et al., 2008). Some studies reported synergic effects of RSV to improve age‐related cognitive decline in AD and PD (Sun, Wang, Simonyi, & Sun, 2008). These studies promise new opportunities for therapeutic agents with improved bioavailability for neurodegeneration treatments.

### 2.4 Resveratrol and cancer

Rates of death by cancer are increasing worldwide with an estimated 12 million deaths in 2030 (Society, 2007). The number of death related to cancer will be expected to be doubled each year in the future. Changes in diet and lifestyle are two main environmental factors causing cancer. Recently, the relationship between dietary factors and cancer prevention in different tissues has been investigated. Many compounds have been shown to be efficient for carcinogenesis prevention in animal models and humans as well. Several studies suggest that natural polyphenol compounds have a great effect on cancer protection (Howe et al., 1992). These compounds that are secondary metabolites in plants and polyphenol diet have shown promising against cancer (Lall, Syed, Adhami, Khan, & Mukhtar, 2015). RSV as a polyphenolic compound in plants has a broad protective activity against several types of cancer. Broad in vivo and in vitro investigations have performed in different types of cancer in recent years. PubMed (PubMed Central) was searched with resveratrol and cancer keywords resulted in 2,567 article in this regard. Abundant documents demonstrate the inhibitory effects of RSV on cancer cell growth, cell cycle, and apoptosis. In addition, RSV has the ability to induce differentiation in some cell lines (Wang et al., 2010). Nowadays, there is a growing attention to applying naturally occurring compounds as preventive and therapeutic agents in cancer management. Due to chemo‐resistance and lack of efficiency of common drugs used in cancer treatment, the development of new nature‐derived compounds with high efficiency and low toxicity seems to be essential for management of this dangerous disease (Fresco, Borges, Diniz, & Marques, 2006). There had been a low interest in the therapeutic index of RSV until the 1990s. In 1997, Jang et al. (1997) conducted a study in the field of protective effects of RSV on the skin cancer in mice model of tumorigenesis. After that, many papers published that represent an increasing interest to this compound in the prevention and treatment of cancer and numerous in vitro and in vivo studies were carried out about anticancer properties of natural compounds such as RSV. Results of RSV effects as anticancer agent indicated inhibitory effects of it in three stages of cancer including initiation, progression, and metastasis. In these studies, it was clear that RSV cause cell cycle arrest and finally apoptosis of tumor cells (Dong, 2003). In addition, many of anticancer properties of RSV were related to its antioxidation effects. Resveratrol as an antioxidant agent can lead to DNA damage prevention that is a major cause of tumor formation. RSV‐based chemoprevention and treatment of cancer consist of cellular and molecular mechanisms. One of the anticancer mechanisms of RSV is through induction of apoptosis (Kalra, Roy, Prasad, & Shukla, 2008; Murakami, Matsumoto, Koshimizu, & Ohigashi, 2003; Nakagawa et al., 2001). RSV causes apoptotic effects in various cell lines including JB6, HL‐60 cells, and various human cancer cell lines (Ahmad, Adhami, Afaq, Feyes, & Mukhtar, 2001; Bode & Dong, 2000; Huang, Ma, Goranson, & Dong, 1999; She, Bode, Ma, Chen, & Dong, 2001).

#### 2.4.1 Resveratrol functional mechanisms in cancer prevention

**Figure 8:**
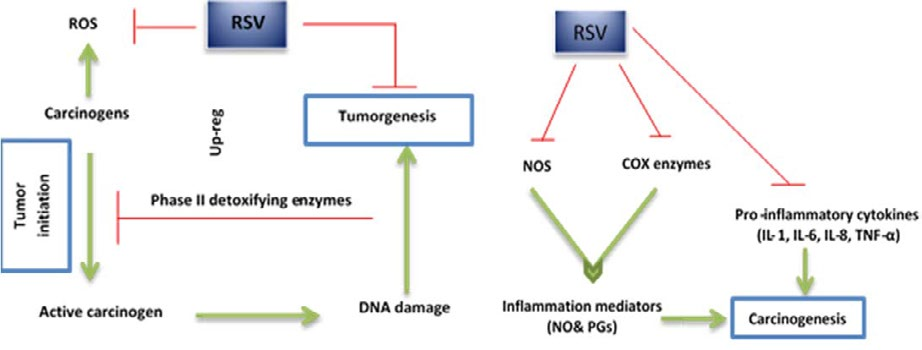
Cancer protective property of resveratrol and its probable mechanisms

Cancer prevention property of RSV revealed in multiple studies; nevertheless, accurate anticancer mechanisms of RSV remained unknown. Induction of apoptosis is the main approach to cancer prevention as well as therapy. Induction of apoptosis by RSV reported in several in vitro cell culture and in vivo studies. P53 is a tumor‐suppressor gene and plays an critical role in resveratrol‐induced apoptosis (McCarthy, Symonds, & Van Dyke, 1994). However, RSV induces P53‐dependent transcriptional activation (Mahyar‐Roemer, Katsen, Mestres, & Roemer, 2001). In some of the tumor cell lines, it induces apoptosis in a P53‐independent manner (Mahyar‐Roemer et al., 2001). Clément, Hirpara, Chawdhury, and Pervaiz (1998) showed the apoptosis inducing via a CD95‐CD95L pathway in HL‐60 and T47D cells. In addition, RSV makes apoptosis induction through inhibition of mitochondrial ATP synthase. Both of activation of pro‐apoptotic and anti‐apoptotic molecule inhibition by RSV supported in recent studies (Kim, Rhee, Park, & Choi, 2003; Zhou, Chen, Wang, Cai, & Du, 2005). On the other hand, RSV decreases the prostaglandins (PGs) expression by inhibition of COX‐2 enzyme, which catalyzes arachidonic acid conversion into prostaglandins. PGs and NO play important roles in cell proliferation (Sanders, McMichael, & Hendrix, 2000) and angiogenesis (Bertelli et al., 1996), which lead to tumor growth and metastasis. Modulation of detoxifying enzymes (CYPs) (Baur & Sinclair, 2006), inhibition of protein kinase C (PKC) (Subbaramaiah et al., 1998), BCL‐2 (Kalra et al., 2008), and cell cycle regulators (Reagan‐Shaw, Afaq, Aziz, & Ahmad, 2004) are other chemoprevention effects of RSV. Overall, a myriad of studies has shown that several signaling pathways and various RSV targets including CD95 (Gao et al., 2002; Wenzel, Soldo, Erbersdobler, & Somoza, 2005), NF‐κβ (Li et al., 2010), Wnt (Parekh, Motiwale, Naik, & Rao, 2011), SIRT‐1, and PI3K/AKt (Kucharczak, Simmons, Fan, & Gélinas, 2003) involved in RSV‐induced anticancer effects. Tumor infraction and angiogenesis suppression are strategies against cancer spreading. Investigations suggest that RSV may act as an anticancer agent to inhibit angiogenesis through affecting hypoxia‐inducible factor‐1 alpha (HIF‐1α) and vascular endothelial growth factor (VEGF) in different cancer cells in vitro (Garvin, Öllinger, & Dabrosin, 2006; Zhang et al., 2005). Park et al. (2007) reported RSV‐mediated debilitation of LPA‐induce pathways of tumor migration in ovarian cancer cells. Diminishing of matrix metalloproteinase expression by RSV and concomitant inhibition of diverse cancer cells invasions concluded in several studies. In addition, RSV oligomers and derivatives have important biological actions in cancer prevention (Li et al., 2009). Figure 8)

### 2.5 Resveratrol and kidney disease

**Figure 9:**
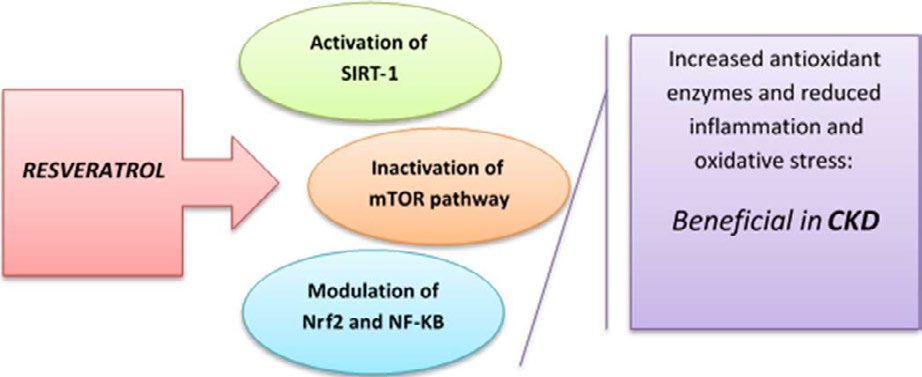
Beneficial effect of resveratrol in CKD by its anti‐inflammation and antioxidant properties

Kidney disorders usually occur due to oxidative stress and inflammation. Polycystic kidney disease (PKD) is an autosomal dominant kidney disorder (Wilson & Goilav, 2007) with mutations in polycystin‐1 (PC1) and polycystin‐2 (PC2) encoding genes (i.e., PKD1 and PKD2). These mutations are initiator cysts formation and progression of disease (Zoja et al., 2014). Some of the pro‐inflammatory factors are identified in cyst fluid or urine samples of PKD patients (Ta, Harris, & Rangan, 2013). Recently, it has been shown that inflammation and cytokine signaling play a significant role in PKD pathogenesis. For example, high concentrations of inflammatory factors (chemokines and cytokines) have been shown by Gardner et al. in kidney patients (Swenson‐Fields et al., 2013). Anti‐inflammatory and antioxidant properties of RSV by inducing antioxidant enzymes production (Spanier et al., 2009) and modulating nuclear factors involved in the inflammation‐oxidative stress cycle (Palsamy & Subramanian, 2011) and established in numerous studies; therefore, it can act as a novel therapeutic agent in kidney disease treatments (Kim, Cha, & Surh, 2010). Wu et al. (2016) demonstrated that RSV acts as an anti‐inflammatory substance, which delayed PKD progression through attenuation of NF‐κB‐induced inflammation. CKD, also known as chronic renal disease, is a progressive loss of kidney function. Of CKD^’^s risk factors, inflammation and oxidative stress are most important (Stenvinkel, 2010). Given that inflammation and oxidative stress are related to CKD pathogenesis, RSV attracted specific interest in its treatment. In 2013, Liang et al. suggested that resveratrol inhibits oxidative stress and renal interstitial fibrosis in mice (Liang, Tian, Han, & Xiong, 2014). In addition, polyphenol supplementation showed that antioxidant activity and lipid profile improvements in hemodialysis patients (Castilla et al., 2008; Yiu, Chen, Chang, Chiu, & Lin, 2010). However, no study has been developed to investigate resveratrol effects in CKD patients, although beneficial effects of RSV could be hopeful in these patients (Figure 9) (Saldanha, Leal Vde, Stenvinkel, Carraro‐Eduardo, & Mafra, 2013).

### 2.6 Hepatic effects of resveratrol

Liver disorders include several maladies such as inborn metabolic disease, alcoholic cirrhosis, viral hepatitis, and drug‐induced hepatotoxicity. The liver disease remains a major cause of morbidity and mortality with significant economic and social costs. Several novel approaches are currently being studied which may provide a better therapeutic outcome for sufferers. The use of naturally occurring polyphenols, some of them obtained from the dietary sources, in the amelioration of illnesses have recently gained considerable popularity (Bishayee, Darvesh, Politis, & McGory, 2010). These agents, having antioxidant and anti‐inflammatory properties, provide a safe and effective means of ameliorating chronic diseases. RSV, a grape polyphenol, has shown a considerable promise as a therapeutic agent in the treatment of the aforementioned liver ailments (Bishayee et al., 2010). The use of RSV supplements with therapeutic and protective potential has investigated in hepatic disease models. Several preclinical and clinical studies have demonstrated the therapeutic usefulness of RSV in several chronic illnesses such as arthritis, diabetes, neoplastic, and neurodegenerative diseases (Saiko et al., 2008). Recently, the studies have shown that RSV has much therapeutic potential on liver disorders. RSV significantly decreased lipid accumulation and apoptosis and provided liver protection against chemical and alcohol‐induced injury. It is able to improve glucose metabolism and lipid profile and decrease liver steatosis. RSV improved hepatic lipid accumulation and progression of nonalcoholic steatohepatitis through down‐regulation of inflammatory signaling pathways (Ji, Wang, Deng, Li, & Jiang, 2015). In a recent study, it was found that mice fed with a methionine–choline‐deficient diet and receive daily intra‐gastric administration of RSV (100 or 250 mg/kg body weight) resulted in reduction of hepatic steatosis and inflammation, while the curative effects of RSV were not seen in the improvement of steatohepatitis (Heebøll et al., 2015). In another study, the findings have shown that daily consumption of RSV with a dose of 500 mg for 12 weeks improved nonalcoholic fatty liver disease in a clinical trial (Faghihzadeh, Adibi, Rafiei, & Hekmatdoost, 2014). The bases for the beneficial effects of RSV are unclear yet. RSV has direct antioxidant effects but also stimulates the expression of antioxidant enzymes and the activity of SIRT‐1 and adenosine monophosphate AMPK both of which have major effects on glucose and fat metabolism and may play a role in aging.

### 2.7 Resveratrol and ophthalmic disease

#### 2.7.1 Glaucoma

Glaucoma is a multifactorial neurodegenerative ocular disease which characterized by progressive apoptosis of retinal ganglion cells (RGCs) (Abu‐Amero et al., 2016; Foster, Buhrmann, Quigley, & Johnson, 2002). Oxidative stress (Tezel, 2006) and mitochondrial dysfunction (Kong, Van Bergen, Trounce, & Crowston, 2009) are damaging factors in glaucoma, and underlying mechanisms are under investigation still. Progressive loss of RGCs leads to increasing level of IOP (Abu‐Amero et al., 2016). Yet devaluation of IOP is impressive procedure to treat the disease, and other ways such as laser trabeculoplasty and surgery may be used to slow the disease progression. Efforts to introduce novel potential drug targets are in focus of research. Luna et al. studied RSV effects on glaucoma markers in trabecular meshwork cells. In this study, the authors reported decreased production of iROS and inflammatory factors (IL‐1α, IL‐6, IL‐8, and ELAM‐1) (Luna et al., 2009). The results of the recent studies have shown the antioxidant potency of RSV in trabecular meshwork cells. It is believed that neuroprotective drug from RGCs against apoptosis could be an effective way in the management of glaucoma. Pirhan et al. (2016) studied the effects of RSV and riluzole on the survival of RGCs in rats. They concluded that combined therapy results in high RGCs protection. The detailed molecular mechanisms underlying riluzole and RSV neuroprotective effects are not clear. Another study using young–adult Thy1‐yellow fluorescent protein (YFP) and C57BL mice confirmed previous study observations which referred based on dendrite RGCs protection role of RSV (Pirhan et al., 2016). Razali and colleagues performed reduction in IOP using the topical use of trans‐resveratrol induced in SIOH rat models. The study suggested that repeated topical use of trans‐resveratrol during 21 days in SIOH rat; maintained IOP reduction, which was related to increasing of aqueous MMP‐2; improvement of retinal morphology; and amendment of the retinal condition (Razali et al., 2015). In addition, trans‐resveratrol‐induced IOP reduction also is important in POAG eyes (Clark & Wordinger, 2009); though, these results need to be certified in human studies. One of the factors that contribute to glaucoma pathogenesis is the role of mitochondrial dysfunction. In this regard, Chen and colleagues evaluated RSV‐induced mitochondrial biogenesis in retinal ganglion cell line RGC‐5. In this study, the authors have shown evidence about the role of RSV to attenuate serum deprivation‐induced elicited RGC‐5 cell death through the SIRT‐1‐dependent PGC‐1α subcellular translocation; therefore, they suggested promoting mitochondrial biogenesis in order to mitigate glaucomatous retinopathy (Liu et al., 2013).

#### 2.7.2 Diabetic retinopathy

Diabetic retinopathy, the most common diabetic eye disease, occurs when the blood vessels of retina change. Sometimes, these vessels swell and leak fluid or even close off completely. In other cases, abnormal new blood vessels grow on the surface of the retina. RSV supplementation has recently been studied in diabetic rat models (Soufi, Mohammad‐Nejad, & Ahmadieh, 2012). In this study, the supplemented rats were compared with diabetic and non‐diabetic controls. Authors reported that resveratrol‐induced suppression of eNOS in the eyes of diabetic rats. eNOS is related to inflammation in chronic diabetes and vascular neovascularization (Yar et al., 2012). Evaluation of the RSV effects on vascular damage and induction of VEGF in the retinas of mice with induced diabetes have shown that increasing of vessel leakage and VEGF protein levels are prevented by RSV treatment (Kim, Kim, Roh, Choi, & Cho, 2012). Li, Wang, Huang, and Zheng (2012) have shown that RSV has inhibition effects on endoplasmic reticulum stress (ERS), which is related to retinal vascular degeneration. In another study, the ability of RSV to inhibit RPE cell inflammation caused by hyperglycemia in vitro was evaluated and inhibition of VEGF, TGF‐β1, COX‐2, IL‐6, and IL‐8 accumulations was observed in a dose‐dependent manner in the treated cells with RSV. In addition, PKCB activity reduction was reported in the presence of RSV (Losso, Truax, & Richard, 2010).

#### 2.7.3 Cataract

Cataract is the clouding of the lens of one or both eyes leading to a decrease in vision. Symptoms may include faded colors, blurry vision, and halos around light, trouble with bright lights, and trouble seeing at night. Cataracts are the cause of half of blindness and 33% of visual impairment worldwide. Cataract is most commonly due to aging, but it may also occur due to trauma or radiation exposure and be present from birth. Risk factors include diabetes, smoking tobacco, prolonged exposure to sunlight, and alcohol. Either clumps of protein or yellow‐brown pigment may be deposited in the lens reducing the transmission of light to the retina at the back of the eye. Age‐related cataract is related to prolonged oxidative stress. This condition is a major cause of blindness in emerging countries (Bola, Bartlett, & Eperjesi, 2014). Cataract rat models created using sodium selenite. Doganay, Borazan, Iraz, and Cigremis (2006) established the RSV role in selenite‐induced cataract preventions. The study reported that RSV treatment leads to increasing levels of reduced glutathione (GSH) in rat lenses and erythrocytes (Doganay et al., 2006). High levels of GSH cause protection against oxidant damaging in lens (Hightower & McCready, 1991). In addition, malondialdehyde (MDA) as a lipid peroxidation marker decreased in a resveratrol‐treated rat. These studies have shown the potential potency of RSV in the selenite‐induced cataract formation (Doganay et al., 2006). Recently, Wang et al. evaluated protective effects of RSV on lens epithelial cell apoptosis in diabetic cataract rats. In this study, a rat model of diabetic retinopathy created by intraperitoneal injection of STZ. The results indicated that RSV could control the progression of cataract in diabetic rat efficiently in a dose‐dependent manner, using caspase‐3 expression level apoptosis ratio as an apoptotic index. The outcomes showed the protective effects of RSV on epithelial cell apoptosis prevention in a dose‐dependent manner in diabetic cataract rat lens. It is noteworthy that regulatory mechanisms of RSV in lens epithelial cell (LEC) is unclear and need to be investigated in more detail. Overall, RSV has the potential to play a role in the treatment of a range of retinal conditions and further clinical trials need to determine the RSV effectiveness as an ocular nutrition supplement.

### 2.8 Antimicrobial and antiviral effects of resveratrol

Antiviral effects of RSV have been investigated in several human and animal viruses including influenza A virus (Palamara et al., 2005), Epstein‐Bar virus (Chen, Qiao et al., 2012), herpes simplex virus (Chen, Qiao et al., 2012), HIV (Clouser et al., 2012), and hepatitis C virus (Nakamura et al., 2010). Results from most of these studies indicate that RSV can prevent inhibiting protein synthesis and thus inhibit virus proliferation (Yang et al., 2015). In summary, in spite of inhibiting protein synthesis, other major mechanisms of RSV against viral infections include the following: (a) signaling pathway inhibition and (b) inhibition of gene expression. In 2015, Mora‐Pale et al. examined the antimicrobial activity of two isomers including resveratrol–trans‐dihydrodimer and Pallidol (enzymatic oligomerization of plant polyphenol RSV in vitro). Authors observed the antimicrobial activity of resveratrol–trans‐dihydrodimer against Gram‐positive bacteria (*Bacillus cereas*,* Listeria monocytogenes*,* Staphylococcus aureas*) and against Gram‐negative *Escherichia coli* with minimal mammalian cells (HepG_2_ cell line) cytotoxicity. In this study, Pallidol had no antimicrobial activity against all above‐mentioned strains. However, authors performed transcriptomic analysis and detected decreasing expression of ABC transporters, genes related to cell division and DNA binding proteins. In addition, flow‐cytometric analysis of treated cells implied membrane potential disruption and DNA synthesis inhibition through blocking of DNA gyrase activity. According to the literature, RSV exhibits the most potent inhibitory activity on *Helicobacter pylori* infection under natural condition (Murano et al., 2005). Chan (2002) evaluated the antimicrobial activity of RSV against bacteria and dermatophytes as skin infection agents and indicated the anti‐fungal effects of RSV. However, the author suggested that resveratrol may have also potential novel applications in diabetic wounds treatment. Hwang and Lim (2015) have shown an antibacterial activity of RSV against *E. coli* and suggested that RSV exerts anti‐*E. coli* activity through suppression of ftsZ expression (an essential cell division protein in prokaryotes) and Z‐ring formation (contractile ring in bacterial cell division).

### 2.9 Leishmanicidal effects of resveratrol

Leishmaniasis is a neglected disease and a public health problem that affects 98 countries. Over 350 million people are at risk, and approximately 0.9–1.2 million new cases of leishmaniasis occurs per year (Alvar et al., 2012). During blood feeding by female sandflies, metacyclic promastigotes are regurgitated. These promastigotes are then phagocytosed by cells at the site of the bite. Once inside the host cells, metacyclic promastigotes transform into amastigotes, which can survive and replicate inside phagolysosomes (Veras & Bezerra de Menezes, 2016). The first‐line drugs for leishmaniasis are pentavalent antimonials. Alternative treatments are amphotericin B, pentamidine, and paramomycin. All of these drugs have high toxicity, low efficiency, side effects, and high costs (Barrett & Croft, 2012; Berman, 2003). For these reasons, the search for new drugs discovery is required for leishmaniasis management. Recently, several natural compounds attracted interest of scientists in leishmaniasis. There are only very few studies about leishmanicidal effects (each of leishmanial species) of RSV and its analogs (four study). First time, Kedzierski et al. investigated resveratrol and it is hydroxylated (3,4,4′,5‐tetrahydroxy‐trans‐stilbene) analog for the cause of cutaneous lesion (leishmania major), which the findings of its study indicated antileishmanial effects of RSV on extracellular (promastigotes) and intracellular (amastigotes) forms and also the analogs of RSV only affected the promastigote form (Kedzierski, Curtis, Kaminska, Jodynis‐Liebert, & Murias, 2007). Nevertheless, previous study (Kedzierski et al., 2007) for antileishmanial effects of RSV, Lucas and Kolodziej (2013), has shown RSV antipromastigotes activity at 153.2 μM, inhibition effect on NO production, and induction of apoptosis in noninfected macrophages. In addition, the results demonstrated that antileishmanial activity of RSV was due to cytotoxic effects on host cells not because of its antiparasitic properties. In addition, Cytotoxic effects of RSV on noninfected J774‐G8 and BMMΦ cells have been shown using FASC analysis, MTT assay, and microscopic evaluation also have been shown, although authors mention that J774‐G8 is a cancer cell line and cytotoxic effect of RSV on this cell line may be arising from chemoprotective properties of RSV (Lucas & Kolodziej, 2013). Furthermore, other studies in human models need to be evaluated. Ferreira et al. studied the effects of RSV and RSV + AMB against *leishmania amazonesis*, and they indicated the antipromastigote (IC_50_ =27 μM) and antiamastigote (IC_50_ = 42 μM) effects of RSV. RSV with AMB showed synergism for amastigotes of this Leishmania species. Cell cycle analysis showed that RSV increased promastigotes in G_0_/G_1_ phase, reduced mitochondrial potential, and elevated choline peak and CH_2_‐to‐CH_3_ ratio in NMR technique (Ferreira et al., 2014). All of these results indicate which parasite dies under study condition. In recent year, leishmanicidal effect of four synthetic trans‐resveratrol analogs has performed (Passos, Ferreira, Soares, & Saraiva, 2015). The most important result of this study was that piceatannol analog was the more promising agent for the treatment of leishmaniasis. Complete results of this study are shown in Table 2. All of these studies suggested the RSV and RSV with AMB could be a novel potential therapy in leishmaniosis control and merit further investigation in future.

**Table 2:** Effect of resveratrol and its derivatives on the *leishmania*

Leishmanicidal effect of synthetic trans‐resveratrol analogs (Passos et al., 2015)		
Resveratrol analogs	IC_50_ promastigotes (μM)	IC_50_ amastigotes (μM)
Pterostilbene	17.7	32.2
Piceatannol	65.0	45.0
Polydatin	95.5	29.0
Oxyresveratrol	65.0	30.5
Piceatannol analog also: Increased fivefold the promastigotes in the sub‐G_0_ stage and decreased 1.7‐fold in G_0_–G_1_ phase Changed mitochondrial membrane potential (ΔΨm) Increased number of annexin‐V‐positive promastigotes		

*Note*

IC_50_: inhibitor concentration.

## 3 CONCLUSION

Natural compounds for prevention and treatment of different diseases especially cancer and chronic diseases attracted more interest in recent years. Resveratrol is a natural polyphenol that is present in different human dietary sources. Investigations have shown that resveratrol supplementation has many beneficial effects for prevention and treatment of a variety of diseases with different pathophysiological mechanisms. A wide range of studies pertaining to beneficial effects of resveratrol is conducted in vitro and animal model experiments. It has the capacity to interact with several molecular targets and has been found non‐toxic in these models. On the other hand, these trials must be conducted with optimization of resveratrol bioavailability, dosage, and safety profile in a different population. Yet, there are limited clinical trials and extensive human studies with large sample size are needed in order to determine the potential effects of resveratrol for therapeutic use from bench to bed.

## CONFLICT OF INTEREST

The authors declare that they have no conflict of interest.

## ETHICS APPROVAL AND CONSENT TO PARTICIPATE

Not applicable.

## HUMAN AND ANIMAL RIGHTS

No animals/humans were used for studies that are the basis of this review.
